# Cardiovascular Disease Risk Among the Poor and Homeless – What We Know So Far

**DOI:** 10.2174/157340309787048086

**Published:** 2009-01

**Authors:** Charlotte A. Jones, Arjuna Perera, Michelle Chow, Ivan Ho, John Nguyen, Shahnaz Davachi

**Affiliations:** 1 University of Calgary, Calgary, AB, Canada; 2 Calgary Health region, Calgary, AB, Canada

**Keywords:** Homeless, cardiovascular diseases, healthcare.

## Abstract

Homelessness [and poverty] is rapidly escalating across North America and is associated with dire implications for public health and our health care systems. Both are compelling states of existence affecting all ages, ethnicities and both genders. Homelessness frequently evolves through a complex interaction of factors that are both internal and external to the individual themselves. Once homeless, equitable access to both preventative and remedial health care is lacking and is associated with a higher than average burden of cardiovascular disease [CVD] risk factors, morbidity and mortality and is accompanied by disproportionately high health care costs. The emergence of limited, small scale programs aimed at addressing the unique health and social needs of the homeless is encouraging. However, there has been inadequate commitment at the National, State or Provincial and local levels to implement policies and dedicate funding and resources to the expansion of such “individual level” interventions into comprehensive programs that deliver sustainable, integrated prevention and services, especially with regard to CVD. The long-term solutions that address the links between homelessness and CVD lie in preventing homelessness and reversing the trends in our health care system that create disparities for lower socioeconomic status [SES] and homeless individuals.

## METHODS

	The primary purpose of this article is to review the available literature detailing the relationships between poverty, homelessness and cardiovascular disease. Secondarily, some of the more current efforts aimed at reducing disparities in CVD care of the poor and homeless will be discussed. Relevant English language articles were identified by searching the Cochrane Database of Systematic Reviews [1996-present], MEDLINE [1966-present], EMBASE [1980-present], CINAHL [1982-present], and BIOSIS previews [1980-present]. Searches were supplemented by scanning bibliographies of included articles, review articles, article citation listings and relevant websites. The literature search was performed in February and March 2008. Search themes included homelessness and health care: Boolean operator “or” was used to combine exploded versions of subject headings including homelessness, health care, poverty, cardiovascular diseases and myocardial infarction [MI], heart failure, stroke, transient, cerebrovascular accident [CVA], cardiovascular procedures, poverty, SES and disparities.

### The Growing Challenge

	Homelessness is a serious and rapidly escalating dilemma in both rural and urban North America. Recent estimates [1, 2] suggest that homelessness affects up to an estimated 800,000 persons on any one week in the United States. Furthermore, between 3-5 times as many people experience homelessness over a year’s time as are homeless on any one particular day [1, 3]. Cities in both Canada and the United States such as Toronto [4, 5],Calgary, Philadelphia, and New York, [5] have reported that between 1 and 1.3 percent of their total population have used a shelter in the previous year. Furthermore, as the number of homeless increase, many inner city shelters are becoming overwhelmed and an increasing number of homeless sleep out in the streets rendering most shelter counts of the homeless as significant underestimates of the problem. For example, the overall homeless count in Calgary [6] increased by 32% between 2004 and 2006. This figure not only represented a 15.7% increase in those enumerated in shelters, emergency or transitional beds, but also a 237% increase in those enumerated who were on the streets that would otherwise have been missed by traditional homeless or “shelter”-based counts. 

	Becoming homeless involves a dynamic interplay between personal and environmental/social elements [7]. Individual factors including adverse childhood experiences, lack of education, inadequate or no job skills, family breakdown, personal disability, mental illness or substance abuse may collide with societal factors such as poverty, escalating housing costs, unfavorable labor market conditions, limited public benefits, racism and discrimination resulting in an increased likelihood of becoming homeless and suffering from ill health, which in turn makes it more challenging to rise out of the homeless state. In “booming” economies such as that in Calgary Alberta, there has been a massive influx of people. Housing costs have increased far more rapidly than wages and the potential for becoming or being on the verge of homelessness has become staggering. In Calgary, [population one million: 2007] over 58,000 households are considered “one paycheck or crisis away from homelessness” [8].

	Health problems associated with homelessness are documented throughout the literature: nearly 40% of homeless individuals are reported to have some type of chronic disease [9] including increased rates of cardiovascular and infectious diseases [10-15] along with excessive rates of substance [tobacco, alcohol and cocaine] abuse [16]. Additionally, psychotic and affective disorders are common, with prevalence rates ranging from between 10% to 13% and 20% to 40%, respectively [17]. Individuals lacking stable housing are more likely to use the emergency department rather than an ambulatory care clinic as their regular source of care [18]. In one study, homeless individuals made 20% to 30% of all adult emergency department visits [19]. When homeless individuals finally present for medical attention, they are more likely than the general population to have multiple medical problems, and often their illnesses have progressed to a more severe stage than normally seen. This helps to explain why homeless patients are admitted to inpatient units 5 times more often and have average lengths of stay that are longer than those who were not considered homeless [20,21].

### Impact of Homelessness Upon All Cause and Cardiovascular Mortality

Mortality rates of the homeless in North America are at least 3-5 times greater than that seen in the general populations of Canada and the United States [22-24]. Cardiovascular diseases are a major cause of mortality in homeless adults between 45 and 64 years old and are three times more common in the homeless aged 25 to 44 years when compared to an age-matched general population [22-24]. Increased CV mortality rates in the homeless are attributable to a complex interplay between traditional and unique or less traditional risks. These risks include the pervasive, immeasurable psycho-social stressors of the daily battle for the necessities of life including food, shelter, safety which along with a decrease in diagnostic, preventative and remedial care results in an increased prevalence of and/or poorer control of the traditional risk factors and other co-morbidities. 

Poverty has been deemed one of the major societal determinants of cardiovascular disease worldwide [25]. Cardiovascular disease evolves from material deprivation, excessive psychological stress, anger, mental illness, the adoption of unhealthy coping behaviors such as alcohol, drug and tobacco abuse along with challenges involving ethnicity, education and employment. Poverty and lower socioeconomic status [SES] have been associated with inadequate primary and preventative health care [independent of ethnic origins], regardless of housing status. Diez-Roux *et al. *[26, 27] demonstrated that in the United States, living in a deprived neighborhood [using four measures: income, occupation, education and house value] was associated with an increased prevalence of CHD and its risk factors and greater than a 70% and a 30-50% increase in the risk of CVD in Caucasians and African Americans, respectively. Socioeconomic deprivation has been identified as an independent risk factor for both admission and readmission to hospital for heart failure [28].

Multiple studies [29-36] from around the globe have correlated the strength of the relationship between lower SES and low neighborhood socioeconomic environment with increased cardiovascular morbidity and mortality. Low SES has been associated with an increased prevalence of uncontrolled CVD risk factors [as above] and prevalence and incidence of angina, as well as a decrease in access to and utilization of evidence-based medications and cardiac care during and post hospital discharge. Increased rehospitalization after MI and short-term and one year CVD mortality have also been shown to be related to lower SES. 

With the exception of smoking and an unhealthy diet, it is unclear whether the prevalence of traditional CVD risk factors in homeless people is greater or less than in the general population. However, there is general agreement that there is a high prevalence of undiagnosed and/or inadequately managed risks such as hypertension, diabetes and cholesterol disorders in the homeless compared with the general population  [37-39].

Szerlip [39] undertook a retrospective chart review of 100 randomly selected patients seen in a homeless clinic in New Orleans, and using 200 matched housed patients attending an inner-city clinic, looked for differences in the prevalence of hypertension, diabetes, cigarette smoking and hypercholesterolemia. They found hypertension in 65% of the homeless and in 52% of the housed [P < 0.05; odds ratio 1.78 [95% CI 1.09-2.9]]. Smoking was by far more common in the homeless [75% vs. 57% [P, 0.005; OR 2.22 [95% CI 1.27-3.88]], while no difference was noted in the prevalence of diabetes or elevated total cholesterol. Lee *et al. *[37] were the first to explore in detail the CV risk factors both by survey interview recall and physical measures in a sample of 202 randomly selected single adults from 17 of 35 shelters in Toronto, Ontario between May 2002 and March 2003. They found that the prevalence of smoking was 78% [81% among men and 57% among women] [95% CI, 72%-84%]. When standardized morbidity ratios [SMR] for comparison with an age- and sex-matched group of individuals in the general population of Canada were calculated, smoking rates among this homeless population were increased by between 2 and 3 fold. The prevalence of diabetes, hypertension and elevated body mass index were not found to be increased in this population of homeless adults, however they found convincing evidence of decreased awareness and medical treatment of these three potent cardiovascular risk factors. For example, only 33% of the hypertensive homeless cohort was aware of having hypertension and only 17% were taking antihypertensive medications, while 57% of hypertensive individuals in the [Canadian] general population were aware of having hypertension [40] and 34% were taking antihypertensive medications [41]. Furthermore, Lee *et al. *[37] demonstrated that 43% of those with diabetes were shown to have poor glycemic control [defined as a HgA1c of > 8.4%]. Suboptimal diabetes control has also been reported in a different homeless cohort by Hwang and Bugeja [42] who demonstrated that 44% their Toronto cohort with type II diabetes had very poorly controlled diabetes [HgA1c’s > 8.9] compared with around 23% of a population-based sample of adults with type II diabetes from the United States.

However, some studies have suggested that traditional CVD risk factors account for no more than half of the socioeconomic gradient in CVD morbidity and mortality, regardless of housing status [43], implicating the significant contribution of other less traditional and immeasurable risk factors. Excessive alcohol and cocaine abuse is reported in as many as 30% of the homeless, representing prevalent and potent less-traditional CVD risk factors in this population [44, 45]. Diagnosis and treatment of both traditional and non traditional cardiovascular risk factors is inadequate in the homeless even in Canada with its system of universal health insurance, suggesting other factors play a significant role. In the United States where more than 50% of the homeless lack health coverage [1], diagnosis and treatment of their cardiovascular risk factors is even more challenging. 

### Nutrition and the Homeless

	Homeless people eat foods prepared , for the most part, by municipal and charity shelters, drop-in centers, fast – food restaurants, 24 hour convenience stores and from garbage bins. Data on the nutritional intake of homeless people is somewhat limited. As assessed by dietary recall protocols, most studies report a high prevalence of inadequate or imbalanced nutrient, vitamin and mineral intake placing the homeless at risk for nutrition-related disorders and contributing to the increased prevalence of poorly controlled diabetes, hypertension and cholesterol [46], all well established risk factors for CVD. Luder *et al.* [49] revealed that homeless people’s diets are often high in saturated fats and cholesterol and inadequate in essential nutrients, contributing to adverse lipid profiles. Another [50] study of nutritional status among a group of adult homeless women found a similar trend: recommended daily allowances [RDAs] were commonly exceeded for sodium and saturated fats, both of which are independently associated with increased CVD risk [51,52]. Furthermore, if the homocysteine theory of arteriosclerosis [53] proves to be valid, then the vitamin and mineral deficiencies commonly found in the homeless, including vitamins B-6, B-12 and folate could also indirectly increase CVD risk through elevation of homocysteine levels. Although the association between nutrition and CVD risk factors in the homeless is complex given the confounding association between substance abuse, concomitant illness, malnutrition and poor nutrient quality, improving nutritional adequacy via improvements in overall food quality will likely result in a decrease in chronic diseases, including CVD.

### Impact of Psycho-Social Factors on CVD Risks

	Mental illness is frequently considered a CVD risk factor and further, may act as a barrier to optimal cardiac care, thus contributing to increased CVD morbidity and mortality. 

	A retrospective cohort study using the General Practice Research database covering 741 practices in the UK from 1987-2002 concluded that the risk of mortality from coronary heart disease is increased in people with severe mental illness in the 18–75 years age group [54]. Poorer mental health has been strongly associated with lower socioeconomic status and homelessness [55, 56]. Serious mental illness including schizophrenia, major depression, and bipolar disorder may be up to 10 [11% *vs*. 1%] fold more prevalent in homeless people than in the general population [17, 57]. In 2007 the Canadian Institute for Health Information [CIHI] [58] reported that among patients recorded as homeless in 2005-2006, the most common reasons for hospital visits were substance abuse, which accounted for 54 per cent of visits [62 per cent for homeless men and 30 per cent for homeless women], followed by other psychotic disorders [20 per cent of visits] such as schizophrenia [28 per cent for homeless women and 18 per cent for homeless men]. 

	While hypertension prevalence has been reported to be 40% lower among homeless people with schizophrenia than in the general population, the documented rates of hospital admission for complications of hypertension, including cardiomyopathy and congestive heart failure are 1.8 and 1.5 times greater respectively [59], suggesting that there are high rates of undiagnosed or underreported hypertension in this population. Further, those with schizophrenia hospitalized for myocardial infarction are less likely than the general population to receive state of the art medical care including cardiac catheterization, PTCA and CABG [60, 61] and have mortality rates 34% higher than that of the general population. However, of those undergoing cardiac catheterization, rates of PTCA and CABG are similar to those without mental disorders [61]. 

	It has become quite clear as to why mental illness leads to greater cardiovascular disease risk. People with severe mental illness have a much more adverse CVD risk profile. David Osborn and colleagues [54] reported that patients in primary care with severe mental illness were more likely to smoke and to have diabetes, low amounts of HDL cholesterol, and raised Framingham risk scores [a composite of risk for coronary heart disease] than those without severe mental illness. Adjustment for use of psychiatric drugs and socioeconomic factors only partly accounted for the association.

	The clustering of risk factors for coronary heart disease in patients with severe mental illness may be linked to the underlying mental illness. Genetic polymorphisms—e.g., in the α7 nicotinic receptor subunit gene—could make individuals with schizophrenia more vulnerable to nicotine addiction than those without the polymorphism [62]. 

	Furthermore, drugs used to treat severe mental illness come with a wide range of deleterious side effects that increase CVD risk. Atypical antipsychotics have been associated with weight gain (sometimes in excess of 7 kg), increased insulin concentrations, and insulin resistance [63].

	Additionally, people with schizophrenia sometimes have difficulty adhering to their antipsychotic drug regimen: as many as 50% of patients are non-compliant with treatment during their illness [64]. This fact makes it difficult for such individuals to adhere to other therapies, such as antihypertensives or antilipaemic drugs—and comply with lifestyle recommendations, such as diet and regular exercise.

### Impact of SES on Cardiovascular Morbidity and Mortality

	Those of lower SES and the homeless may experience barriers to appropriate cardiovascular disease prevention and care. In addition to poorly controlled CV risk factors, many will delay seeking care and may undergo fewer cardiac procedures, take fewer medications and undergo less post hospital discharge care and follow-up. 

	Studies from Finland [34], Scotland [35], Sweden [36], the United States [30,  33] and Canada [31] have repeatedly demonstrated that low neighborhood income and education are independent predictors of incident CHD, pre-hospital all cause mortality and short-term [during hospital admission] and one year CVD mortality rates post MI. This relationship is thought to be related to the poorer baseline clinical status of lower SES patients [older, female, non-Caucasian, more prevalent tobacco use, poorly controlled diabetes, hypertension, dyslipidemia, prior CVD and heart failure] coupled with delays in seeking medical attention [11]. Some have suggested that these disparities exist regardless of housing status or racial and ethnic categories [11, 31, 32, 65-68]. Meanwhile others [33, 26] suggest that the relationship between household income and cardiac care and mortality is attenuated when data is adjusted for demographic, clinical and quality of care factors. Salomaa *et al.* [34] showed that the adjusted risk ratio of prehospital coronary death was 2.11 [95% CI 1.82-2.46] and 1.68 [95% CI 1.14-2.48] respectively among low income men and women compared to those with high income; likely reflecting the poorer baseline health status and increased delay from onset of symptoms to medical presentation in the lower SES group [regardless of whether they lived in rural or urban neighborhoods]. Delay in presentation after symptom onset and lower patient physician contacts have been reported elsewhere for low SES and homeless patients [35, 69, 70]. Increased mortality has been shown to be directly related to longer [*vs*. shorter] times from symptom onset to fibrinolytic and angioplasty treatment in acute myocardial infarction [reviewed in 71]. Further, increased time to fibrinolytic therapy from hospital or emergency department admission [“door –to –needle” time] likewise has a significant negative impact upon mortality [70, 71]. The greater delays and lower rates of angiography in the lower SES groups, coupled with their higher rates of co-morbidities help to explain the poorer in-hospital survival rates.

	Chang *et al.* [31] recently demonstrated that because lower SES groups suffered an increased rate of emergency room mortality, they appeared to have lower hospital admission rates compared to higher SES groups. However, when this observation was accounted for, hospital admission rates from the emergency departments were the same for all SES groups. This finding of increased emergency room mortality is consistent with the documented higher risk profiles of lower SES groups. Another study of 169,079 [>65 years old] Medicare beneficiaries admitted to one hospital for acute myocardial infarction showed that the poorer patients were less likely to receive aspirin or reperfusion on admission [32] . These findings are somewhat in contrast to those of Chang *et al.* [31], who determined that one year revascularization rates were similar amongst all income quartiles when adjusted for the emergency room department volume [lower SES less likely to visit an emergency department at a high volume, metropolitan, tertiary hospital with interventional facilities]. Interestingly, Atler’s group [65, 72, 73] determined that regardless of whether the hospital had facilities for angiography, revascularization or both, there were longer wait times and lower angiography rates for lower SES groups. However, revascularization rates were found to be the same across SES quintiles for those that underwent angiography [65]. Chang’s group demonstrated a significant interaction between SES and revascularization [P = 0.03], suggesting that the effect of SES was largely confined to the non-revascularized patient. 

	The inverse relationship between SES quintile and increased early [emergency department and /or three month] and one year crude mortality rates has been shown to be independent of the emergency room volume or hospital on-site facilities [31, 65, 72, 73]. However, once again, the relationship with SES and one year mortality was abolished when adjusted for the baseline characteristics of the study participants [less affluent patients were significantly older, more likely to be female, South Asian, reside in rural communities, have diabetes, hypertension and pre-existing heart disease] [73]. These findings were in agreement with Rao’s group [67] who demonstrated a non-significant trend [adjusted hazard ratios and 95% CI of 30-day and 6 month death or MI: 1.3 [.8-2.1] and 1.4 [.9-21] respectively], favoring a disparity in 30-day and six month death or MI between low and high income patients after multivariate adjustment for baseline and cardiac care processes. However, only 7% of Lee’s cohort [37] with self-reported CVD [coronary artery disease, peripheral vascular disease or stroke] reported taking daily aspirin while only 31% with high cholesterol reported taking cholesterol lowering medications. In addition, low SES was associated with a lower likelihood of receiving aspirin or beta-blockers on hospital discharge [32], and cardiac rehabilitation or follow up by a cardiologist [73]- findings that might contribute to increased early and late CVD mortality.

	Given the admixture of traditional and non-traditional risks of the homeless individual, it is unclear how generalizable these findings are to the homeless and whether simple statistical adjustment for traditional risk factors can nullify the “homeless”-effect [non-traditional risk factors] upon CVD morbidity and mortality. However, while studies reporting on low SES or poverty may not pertain directly to the homeless, they may serve to emphasize the contribution of varying disparities on CV health and health care.

### The Homeless and Barriers to Quality Health Care

	Disparities in CVD care appear to arise from a complex interaction between environmental, physical, psychosocial and economic determinants, even under the Canadian system of universal health care. Disparities appear to be influenced by characteristics of the physician as well as the homeless patient [74].

	Much work has been done to investigate the extent to which patient factors contribute to racial, ethnic and socioeconomic healthcare disparities. Major determinants, many of which apply to the homeless, include not only healthcare insurance access but language preference, patient health literacy, mistrust of providers with or without feelings of discrimination, medication non adherence, patient preferences, doubts about self-efficacy [74-81] and refusal, inability or delay in seeking and adhering to treatment. As a result of such factors, many homeless have a very high rate of sporadic and ineffective health care utilization, such as at emergency rooms or walk-in clinics where continuity of care and prevention are not the focus [82, 83]. 

	Data attempting to separate out patient preference from the possibility that the homeless or those of lower SES are less likely to be offered certain CVD treatments are conflicting and incomplete. A cross sectional survey  [84] of 272 black and white volunteer VA Medical center outpatients assessed patient preferences and their contribution to racial differences in revascularization procedures [angioplasty or coronary artery bypass [CABG]. Although racial differences were found to play a small role, multivariate analysis showed that preferences were more closely related to a poorer understanding or familiarity with the procedures by black participants who were less inclined to undergo procedures when compared with the white participants. The authors concluded that along with further research in the area, patients of all races might benefit from improved communication regarding proposed cardiovascular procedures and that health care providers might benefit from support or resources to help increase their cultural competence, understand patients’ language, and better understand health literacy needs.

	Barriers to medication adherence in the homeless may contribute to increased CVD morbidity and mortality. Factors leading to non adherence include challenges around access, storage and ability to follow treatment scheduling  [69]. Barriers to access include financial, system-related challenges regardless of presence or absence of drug plans and barriers to storing or holding on to medications that can be stolen, lost, forgotten or sold to obtain “bus money or cigarettes”. Additionally, it is difficult to follow medication schedules for those with inadequate social support who are unable to manage their own medication, or do not have a safe environment to take their medications [e.g. insulin with its needles and syringes being stolen] [69, 42]. Those suffering from alcohol abuse or mental illness may be less able to follow directions for taking medications (e.g. only at meals), while unpredictable housing and transportation challenges make it difficult to keep medical appointments [69, 42, 38]. Furthermore, many homeless place their health as a lower priority than food, safety and shelter and may not be motivated to appropriately access and adhere to medications [29, 69], particularly for asymptomatic chronic diseases such as hypertension, which inevitably increase their CVD risk.

	Perceived or real negative interactions between patients and health care providers may contribute to disparities by compromising optimal patient care and clinical outcomes. For example, in a very small survey [n=31] carried out at a homeless shelter in Calgary, Alberta  [85], 55% of the respondents stated they had faced discrimination within the health care system due to their homeless status. Of these individuals, 77% stated that they had hesitated to approach health care services because of their homeless status compared to only 21% of those who stated that they had not experienced discrimination. A recent survey of 6,722 adults [86] examining factors in the health care encounter revealed that between 14 and 20% of blacks, Hispanics, and Asians *vs*. only 90% of whites felt they had been “looked down on or treated with disrespect”. Non Caucasians were also more likely than whites to feel they had been treated unfairly because of their race. Similar perceptions of disrespect were noted for those living below the poverty line and those participants that had achieved equal to or less than secondary school level. Respondents reporting being treated with disrespect were significantly less likely to have had a physical examination or optimal care for their diabetes, hypertension or heart disease in the previous year. They were also more likely to report not following doctor’s advice and stated they had delayed seeking attention for a medical problem. Perceptions of disrespect or receiving unfair care are common and may negatively impact patients’ access to optimal health care and likely contribute to current health disparities. 

	Health care provider attitudes and decision making may contribute to disparities in treatment for CVD and reflect poorly on the patient provider relationship. Providers may feel uncomfortable treating homeless or mentally ill individuals  [87] and may not recognize their discrimination or stigmatization of such people. Disparities in health care may thus arise through inadvertent prejudice, stereotyping, clinical uncertainty due to cultural ignorance and referral bias [Reviewed in  74, 88, 89]. A recent study [89] of 344 cardiologists revealed that less than one third were even aware that disparities in cardiac care existed in the American health care system. 

	The homeless may delay seeking help for a multitude of reasons. Along with their multiple and often uncontrolled comorbidities and substance abuse problems, they are prone to be sicker by the time they initially present for care resulting in increased hospitalizations, complications, morbidity and mortality  [75].

	Targeted efforts to reduce cardiovascular risks in the homeless population may seem inconsequential. However, adequate preventative and treatment measures are likely to reduce morbidity and mortality and in doing so, possibly reduce costly emergency department and hospital visits. Encouragingly, given the opportunity, homeless persons have been shown to seek health care for chronic conditions if they have felt it was important to do so [90]. Dedicated collaborative efforts aimed at addressing competing priorities along with preventative and remedial risk factor management in the homeless are now mandated.

### Achieving Equity in Access to Care and Outcomes

	The essential components of success include: ameliorating and preventing homelessness [and poverty], increasing shelter access and food quality, increasing personal safety, improving health care access and prevention and management of chronic disease, mental illness and substance abuse.

	The homeless represent a population struggling under the collective burdens of residential and nutritional instability, poor social networks, educational/skills training deficiencies, personal safety issues and significant levels of substance abuse, mental illness and physical disease. Upstream, the amount of affordable housing determines the rate of homelessness, while the downstream or individual – level risk factors determine who becomes homeless [91]. Although distinct factors, they are clearly inter-dependent issues and must both be addressed in order to make any significant impact on homelessness and its associated health inequities. Successful interventions must combine efforts to improve housing along with physical and psycho-social determinants of health i.e. address both prevention and remediation of homelessness itself. The key elements of success [92] include a multiplicity of strategic partnerships and programs that acting in concert are capable of tracking individuals through the whole system; that include strong jurisdictional commitments [e.g. the community, the health care system, local state or provincial and federal governments]; that provide significant mainstream agency involvement, and that assure mechanisms for continuous system improvement.

	Health care systems and housing sectors must be intricately linked and hold the key to resolving homelessness. The internal and external barriers to primary and preventive health care and cardiovascular care in particular must be addressed. Efforts to prevent and reduce homelessness and improve the overall and CV health of the homeless [7] have been classified into biomedical, educational, environmental, and political strategies.

### Biomedical Health Care

Given the unique and broad array of stressors placed upon the homeless, primary health care programs that actively seek out and follow-up on the homeless individual [aggressive outreach case-management] before they become ill or develop an ongoing co-morbidity have to date shown some successes in the area of mental health and may serve as models to address other chronic diseases such as CVD. For example, intensive community [local shelters or community health centers] interventions such as the assertive community treatment [ACT] model use teams of psychiatrists, nurses and social workers. The teams closely manage a small group of homeless mentally ill patients, aggressively keeping in touch and providing intensive case-base management [93]. ACT intervention patients had fewer psychiatric inpatient days, more days in community housing and greater improvements in their symptoms when compared with the usual care patients.

Despite the complex challenges of homelessness, there are emerging solutions. A program tailored to the unique needs of shelter and homeless populations has been launched in Calgary, Alberta Canada. This pilot program, titled “Chronic Disease prevention and Management Programs [CDM]”, was launched by the Calgary Health Region (a publicly-funded health care system that serves a population of approximately 1 million in Southern Alberta, Canada) [94]. It uses a partnership-based and community development approach. The initial focus of the program will be aimed at the prevention and management of diabetes and will eventually expand to managing other chronic conditions highly prevalent among homeless people. Active engagement of the homeless, the organizations serving them and multiple local, municipal, provincial and national sectors and stakeholders will be critical in identifying the barriers, needs and best strategies related to the physical, mental, social and financial determinants of health among homeless people. If successful, this initiative could serve as a model for delivery of effective chronic disease interventions for socially disadvantaged populations in Canada and elsewhere. 

### Educational and Behavioral Strategies

	Educational and behavioral strategies have been focused on the public, health care providers [HCP], communities, the homeless, and those at risk of homelessness. To aid in HCP patient interactions, trust and communication must be built up. HCP need to become aware of stereotyping and stigmatizing processes and become vigilant in order to avoid any form of discrimination while striving to remain respectful and culturally sensitive to all patients. They need to be supported by the HC system and provided with the education, experience and resources to overcome this barrier to equity in care [88, 89]. The education and support should start at the medical school level continue unabated, to the level of opinion leaders and agencies within the cardiovascular provider community to local, state or provincial and federal governments. The relevance for HCP becomes evident with survey information obtained from the homeless suggesting they feel somewhat discriminated against and may mistrust and delay seeking treatment because of this. Supporting this sentiment and the need for further research in this area, are two studies suggesting that some medical student’s attitudes towards diversity may actually become more negative during medical school [reviewed in 95]. The aim of such education and training should be to increase awareness, respect, understanding and comfort with the disparities and challenges of poverty and homelessness. Training of HCP should consider including mandatory longitudinal experiential learning within medical school [96], residency, and if appropriate, throughout sub-specialty training, nursing, pharmacy, sociology, dietary, kinesiology and psychology curricula. On site longitudinal community experiences will not only represent and provide a continuing source of health care for the homeless but may decrease the rate of poor control of CVD risk factors while decreasing the delay in presentation to emergency departments, increasing access to procedures, and decreasing in- hospital and post-hospital morbidity and mortality.

### Policy and Legislative Strategies

	Environmental strategies directed at improving cardiovascular and other health risks need also be directed at creating supportive social, economic and physical environments to enable the homeless to address issues other than just the basic necessities of food, safety and shelter. Hwang *et al.* provide some suggestions on how diabetes can be managed among homeless populations [42]. A survey based study of 50 homeless participants in Toronto, Canada, found that the most commonly reported difficulties in managing diabetes were related to the diet at shelters, access to medications and supplies, and the coordination of medications with meals. The authors recommended changes in shelter procedures in order to address the scheduling and logistics issues, to provide for safe storage and ready access to medications and supplies, and to establish a secure place for people to self-administer insulin and use glucose-monitoring devices.

	Strategies need to include accessible housing, public health, and quality of shelter food, immigration and crime control. Successful examples usually include commitments by municipal, county and state or provincial governments to long term [5- 10 years] goals to end homelessness. As of 2007, over 300 communalities in the USA have committed to undertake efforts to end homelessness and 180 communities have completed plans to end homelessness [97]. The city of Portland and Multnomah County has become renowned for the successes it has achieved in its 10 year plan to end homelessness [98]. They achieved anywhere from 50-120% of their two year goals by moving chronically homeless into permanent housing, increasing the supply of permanent supportive housing and increasing economic opportunities, while stopping jail discharges into the homeless state. Many other cities in Canada and the United States are following suit and developing plans to end homelessness based on similar principles as those applied in Portland.

## IN CONCLUSION

	Homelessness and increasing poverty are significant and growing problems in North America and worldwide. Homelessness is a complex state of bare existence affecting all ages, and is associated with a higher than average burden of CVD morbidity and mortality in adults. Causes are internal and external to the homeless themselves. Although the emergence of limited programs aimed at addressing and responding to the unique health and social needs of the homeless people is very encouraging, adequate National, State or Provincial and local resources have yet to be dedicated to a carefully thought out comprehensive plan to deliver sustainable, integrated services to the right targets at just the right time. As Woolff [75] states, “it is not the resources for social reform, but the resolve” that is lacking. Much hard work with governments and policy makers, more research and much more knowledge is required in order to reduce the incidence and prevalence of homelessness and its physical and social consequences. The long-term solutions lie in preventing homelessness and reversing the trend in our health care system that creates disparities for lower SES and homeless individuals. 
